# Predictors for the use of traditional Chinese medicine among inpatients with first-time stroke: a population-based study

**DOI:** 10.1186/s12906-020-03037-9

**Published:** 2020-08-06

**Authors:** Wei-Sen Chen, Hung-Chih Hsu, Yi-Wen Chuang, Meng Lee, Kuan-Yu Lu, Yi-Fei Chen, Chien-Min Chen

**Affiliations:** 1 Department of Physical Medicine and Rehabilitation, Chang Gung Memorial Hospital, Chiayi, No.6, W. Sec., Jiapu Rd.,, Puzih City, Chiayi County 613 Taiwan; 2 Department of Physical Medicine and Rehabilitation, Jing Mei Hospital, Taipei, Taiwan; 3 School of Medicine, College of Medicine, Chang Gung University, Taoyuan, Taiwan; 4 Department of Nursing, Chang Gung University of Science and Technology, Chiayi Campus, Chiayi, Taiwan; 5 Department of Natural Biotechnology, Nanhua University, Dalin, Chiayi, Taiwan; 6 Center for Musculoskeletal Regenerative Medicine, Chang Gung Memorial Hospital, Chiayi, Taiwan; 7 Department of Physical Medicine and Rehabilitation, Xiamen Chang Gung Hospital, Xiamen, China; 8 Jinan Rehabilitation Clinic, Tainan, Taiwan; 9 Department of Neurology, Chang Gung Memorial Hospital, Chiayi, Taiwan; 10 School of Traditional Chinese Medicine, College of Medicine, Tzu Chi University, Hualien, Taiwan

**Keywords:** Traditional Chinese medicine, Stroke, National Health Insurance Research Database, Complementary and alternative medicine

## Abstract

**Background:**

Stroke is one of the major causes of death and disability. The treatments that are provided to patients during hospitalization after an acute stroke are very important in stabilizing their medical condition and enabling the recovery of their motor functions. However, limited information is available regarding the use of traditional Chinese medicine (TCM) during hospitalization for first-time stroke patients. The researchers aimed to investigate the factors affecting TCM use and to provide clinicians with comprehensive information on TCM use among first-time stroke inpatients in Taiwan.

**Methods:**

The researchers collected and analyzed data, including patient characteristics, TCM use, and TCM prescription patterns, from the National Health Insurance Research Database in Taiwan for first-time stroke inpatients between 2006 and 2012.

**Results:**

Among the 89,162 first-time stroke patients, 7455 were TCM users, and 81,707 were TCM nonusers. The predictors for TCM use were as follows: age, 45–64 or < 45 years; men; living in a level 2, 4, or 7 urbanized area; insured amount ≥ 576 USD per month; ischemic stroke; hospitalized for first-time stroke for 8–14 days, 15–28 days, or ≥ 29 days; stroke severity index score 0–9 or 10–19; Charlson–Deyo comorbidity index score 0 or 1–2; hospitalization in a regional or community hospital; receiving rehabilitation; and previous experience with outpatient TCM use. An increase in the number of TCM users was observed from 2006 to 2012. Furthermore, 68.8–79.7% of TCM users used acupuncture only, while 17.8–26.1% used both acupuncture and Chinese herbal medicine.

**Conclusions:**

An increasing number of first-time stroke patients have been choosing TCM as a complementary treatment during hospitalization. Moreover, TCM use is associated with demographic, clinical, and socioeconomic characteristics. These findings may help clinicians comprehensively understand the trend and the important factors affecting TCM utilization among patients who are hospitalized due to first-time stroke.

## Background

Stroke is one of the major causes of death worldwide [1]. Poststroke complications and functional impairment may lead to long-term disability [2]. In Taiwan, the incidence and prevalence rates of stroke are 330/100,000 person-years and 19.3/1000, respectively [3, 4], and the national expenditure for stroke is more than 400 million United States dollar (USD) per year [5], thus making it a large healthcare burden.

Moreover, 95% of stroke survivors show significant functional recovery within 3 months of onset [6, 7]. The in-hospital care and treatment for patients with acute and subacute stroke are very important for stabilizing their medical condition and enabling the recovery of their motor functions [8]. Traditional Chinese medicine (TCM) is a safe treatment [9, 10] that has been used for more than 2500 years. Acupuncture and Chinese herbal medicine are the two major forms of TCM therapy [11].

According to the literature, patients who received routine care and TCM had fewer adverse outcomes [12], a lower rate of readmission for circulation-related complications [13], and a reduced risk of mortality [14] during the early stages of stroke recovery. TCM therapy also improves poststroke dysphagia [15], shoulder pain [16], spasticity [17], self-care ability and quality of life [18].

Taiwan’s National Health Insurance (NHI) program was established in 1995, and it covers more than 99% of Taiwan’s population [19]. Medicine and TCM are two large categories of healthcare provided by the NHI [20, 21], and since NHI programs were started, they covered TCM claims for outpatient clinic services but not for inpatient services. Therefore, the costs of using TCM for stroke inpatients are not covered by the NHI, and hence, these costs were completely covered by the patients before 2006.

The government launched an NHI-sponsored national project in 2006; this project was a pilot scheme of the use of TCM for individuals who had been stroke inpatients for 6 months or less after a confirmed diagnosis of stroke [12, 22]. The goal of this scheme was to provide better healthcare to inpatients with acute or subacute stroke. Every hospital with TCM physicians providing outpatient services in Taiwan could join this scheme to provide extra TCM as part of routine care for stroke inpatients.

The present study focused on the following questions. What was the trend of using pilot scheme-supported TCM in these inpatients with stroke? If TCM treatment for acute and subacute stroke inpatients was also covered by the NHI, according to the pilot scheme of TCM experiences, what percentage of stroke inpatients were expected to undergo TCM (acupuncture and Chinese herbal medicine) during their first-time stroke hospitalization? Most importantly, what were the potential factors affecting TCM use among inpatients during their hospitalization for first-time stroke? To answer these questions, the researchers conducted a retrospective longitudinal study to examine the characteristics, trends, and important factors affecting NHI-sponsored TCM use among first-time stroke inpatients using population-based data from the NHI Research Database (NHIRD). The purpose of this study was to provide clinicians with comprehensive information about TCM use.

## Methods

### Source of the data

In Taiwan, the NHIRD comprises deidentified personal data that are available for research purposes. The database that was used contained longitudinal medical information, including the registry for beneficiaries and contracted medical facilities; details of inpatient and outpatient orders; inpatient and outpatient expenditures; International Classification of Diseases, 9th edition, Clinical Modification (ICD-9-CM) diagnostic codes; and procedure codes of all Taiwanese stroke patients between 1997 and 2013. The researchers collected data on first-time stroke inpatients from 1997 to 2012. Because these are longitudinal data of all stroke patients from 1997 to 2013, patients who were admitted in 2012 may have been discharged in 2013. However, the researchers still had information about the next years’ claims, even for patients who were admitted in late 2012. The researchers did not have the 2014 claim information for discharged first-time stroke inpatients who were admitted in late 2013. Therefore, to avoid bias, the study did not include first-time stroke inpatients who were admitted in 2013. The quality and quantity of NHIRD-related studies have been increasing since the first published study in 2000 [23].

### Inclusion/exclusion of study patients

First, the researchers included patients with inpatient claims, including patient admission data, for stroke diagnosis (ICD-9-CM codes 430–435) from 2006 to 2012. Patients with ICD-9-CM codes 430–435 and 438 (late effects of cerebrovascular disease) for inpatient and outpatient claims, respectively, from 1997 to 2005 were excluded because they were not considered first-time stroke patients. Patients admitted to hospitals outside the TCM scheme were excluded. Patients with no definite date of discharge during hospitalization for first-time stroke were also excluded. Finally, 89,162 first-time stroke patients admitted to hospitals that joined the pilot scheme of TCM were included.

**Fig. 1:**
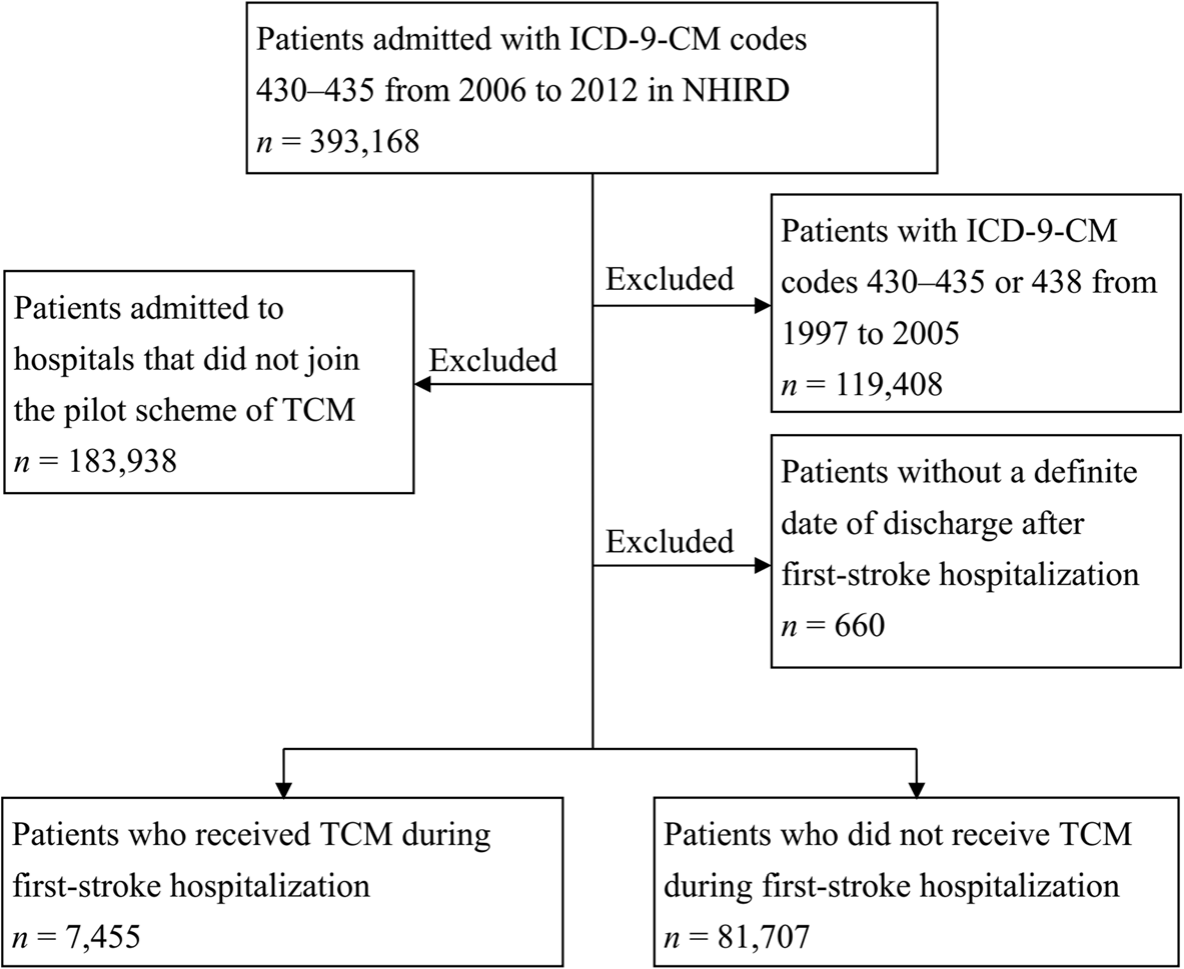
Flowchart illustrating study patient selection. ICD-9-CM, International Classification of Diseases, 9th edition, Clinical Modification; NHIRD, National Health Insurance Research Database; TCM, traditional Chinese medicine

TCM users are defined as stroke inpatients whose claims contain any one of the following codes, especially for the pilot scheme of TCM: supplementary diagnosis fees (P33001), Chinese herbal medicine fees (P33021, P33061), acupuncture fees (P33031, P33032), Chinese traumatology treatment fees (P33041), disease management and care fees (P33051-P33053), and supplementary examination fees (P33071-P33074). Among TCM users, patients who used Chinese herbal medicine and acupuncture were defined using their claim data containing codes P33021 and P33061, and codes P33031 and P33032, respectively. Among these 89,162 patients, 7455 were TCM users and 81,707 were TCM nonusers during hospitalization for first-time stroke. The flowchart illustrating patient selection is shown in Fig. 1.

### Record selection

#### Potential factors affecting TCM use

Information about the factors that potentially affected TCM use via the pilot scheme was available from the NHIRD and was selected for analysis in the study. Demographic factors, including age [24, 25] and sex [24, 25], and clinical factors, such as stroke type [25], length of hospital stay for first-time stroke [22], stroke severity [22, 26], stroke comorbidity [24], hospital accreditation level [26], rehabilitation use during hospitalization [22], and the patient’s previous experience with TCM use [27], were considered relevant. Socioeconomic factors, such as urbanization [24, 25] and insured amount [24, 25], may also possibly affect medical resource utilization among stroke patients.

Recording patients’ information on these factors was based on the following classifications. Age was stratified as < 45 (young), 45–64, and ≥ 65 years (old). Sex was recorded as either man or woman. Regarding stroke type, ICD-9-CM codes 433–435 were recorded as ischemia, ICD-9-CM codes 430–432 were recorded as hemorrhage, and both existing ICD-9-CM codes 433–435 and 430–432 were recorded as combined ischemia and hemorrhage. The length of hospital stay for first-time stroke was stratified as 1–7 (within 1 week), 8–14 (1–2 weeks), 15–28 (2–4 weeks), and ≥ 29 (> 4 weeks) days, and the days of stratification were adopted from a previous NHIRD study on stroke patients [28].

The hospital accreditation level was divided into community hospitals, regional hospitals, and medical centers, which have been used as the 3 hospital accreditation levels in Taiwan [29]. For any given treatment or procedure, higher reimbursements are paid by the Bureau of the NHI to hospitals with a higher accreditation level than to hospitals with a lower accreditation level [30]. However, hospitals with higher accreditation levels are responsible for teaching, training, and research. The accreditation of each hospital is reviewed by the government every 4 years [29, 31, 32].

Rehabilitation use was defined as the presence of claims for physical, occupational, and speech therapy or any combination thereof. Claims for splint orders were not included in the rehabilitation therapy orders. A patient’s previous experience with TCM use was defined as any outpatient claims for TCM in the NHIRD in the past 9 years before the first-time stroke occurrence. For all enrolled patients from 2006 to 2012, the researchers aimed to use previous TCM treatment experience before first-time stroke for as many years as possible, resulting in 9 years of claims. This is because data inclusion was based on the year that the program was started (2006), and NHIRD data were only available starting in 1997, which was just 9 years before 2006.

Sung et al. developed a stroke severity index (SSI) score that is highly correlated with the National Institutes of Health Stroke Scale (.743 correlation coefficient) [33] to represent the stroke severity for patients with stroke in the NHIRD. From the analysis of the multiple linear regression model, the summing constant and each individual coefficient of seven predictive features (osmotherapy, urinary catheterization, nasogastric intubation, airway suction, bacterial sensitivity test, general ward stay, and intensive care unit stay) constitute the SSI score. The SSI score ranged between 4.1043 and 27.1125. A higher score indicates greater stroke severity. The 10-point interval, frequently utilized in studies of stroke inpatients [34, 35] for SSI scores of ≥20, 10–19, or < 10 was used to examine stroke severity in these patients.

The Charlson–Deyo comorbidity index (CCI) score was used to estimate the mortality risk from comorbid diseases. The CCI represents a clinical comorbidity index that was calculated from the sum of assigned weights of 17 different diagnostic categories [36, 37]. A higher CCI indicates greater severity of comorbidity. In this study, the researchers used the diagnosis codes of ICD-9-CM from these diagnostic categories (excluding cerebrovascular disease and hemiplegia) to calculate the CCI score. The CCI scores were stratified as ≥3, 1–2, or 0, which have been previously used for translating the severity of comorbidity of stroke inpatients [34, 35].

The patients’ residential areas were recorded according to 7 urbanization levels. According to a previous study [38], the 359 townships in Taiwan were categorized into 7 urbanization levels (1, most urbanized; 7, least urbanized) based on population density (people/km2), the percentage of the population with college or higher educational levels, the percentage of the population aged over 65 years, the percentage of the population that are agricultural workers, and the number of doctors per 100,000 people (on the basis of Taiwanese census data from the year 2000).

The NHI set the value of 17,280 New Taiwan dollar (NTD) as the lowest monthly salary (tier 1) and 182,000 NTD as the highest monthly salary (tier 55). In this study, however, the monthly insured amount was categorized as < 576 USD (lowest monthly salary) (17,280 NTD; conversion rate of NTD:USD = 30:1) and ≥ 576 USD, considering that most stroke patients were at retirement age and were thus jobless.

#### Trends of TCM use

The number of patients who were admitted to hospitals due to first-time stroke and joined the pilot scheme of TCM in each year between 2006 and 2012 was recorded separately. The annual number of patients who received either of the major types of TCM, i.e., Chinese herbal medicine and acupuncture, was also recorded. The annual rate of TCM use by first-time stroke inpatients was obtained as follows: the total number of first-time stroke inpatients utilizing TCM divided by the total number of first-time stroke inpatients in hospitals that joined the pilot scheme of TCM in each year. The annual utilization rates of Chinese herbal medicine and acupuncture among TCM users with first-time stroke were obtained as follows: the total number of first-time stroke inpatients utilizing Chinese herbal medicine divided by the total number of first-time stroke inpatients utilizing TCM in each year and the total number of first-time stroke inpatients utilizing acupuncture divided by the total number of first-time stroke inpatients utilizing TCM in each year.

### Statistical methods

Statistical analyses were performed using SAS Studio version 3.7. To investigate the potential factors affecting TCM use among these stroke inpatients between 2006 and 2012, univariate associations were analyzed for each potential factor using the chi-square test, and Pearson’s and Spearman’s correlation analyses were used to check for collinearity. TCM was used as a dependent variable and each factor that potentially affected TCM was used as an independent variable in a binary logistic regression. The overall model was used for evaluating the logistic regression model, and the overall model significance for the binary logistic regression was examined using the model coefficients of the Wald chi-square test. The odds ratio (OR), in terms of Exp (β), was calculated for the ratio of the predicted probability of TCM use between two levels of each potential factor. A *P* value of < 0.05 was considered statistically significant.

## Results

**Fig. 2:**
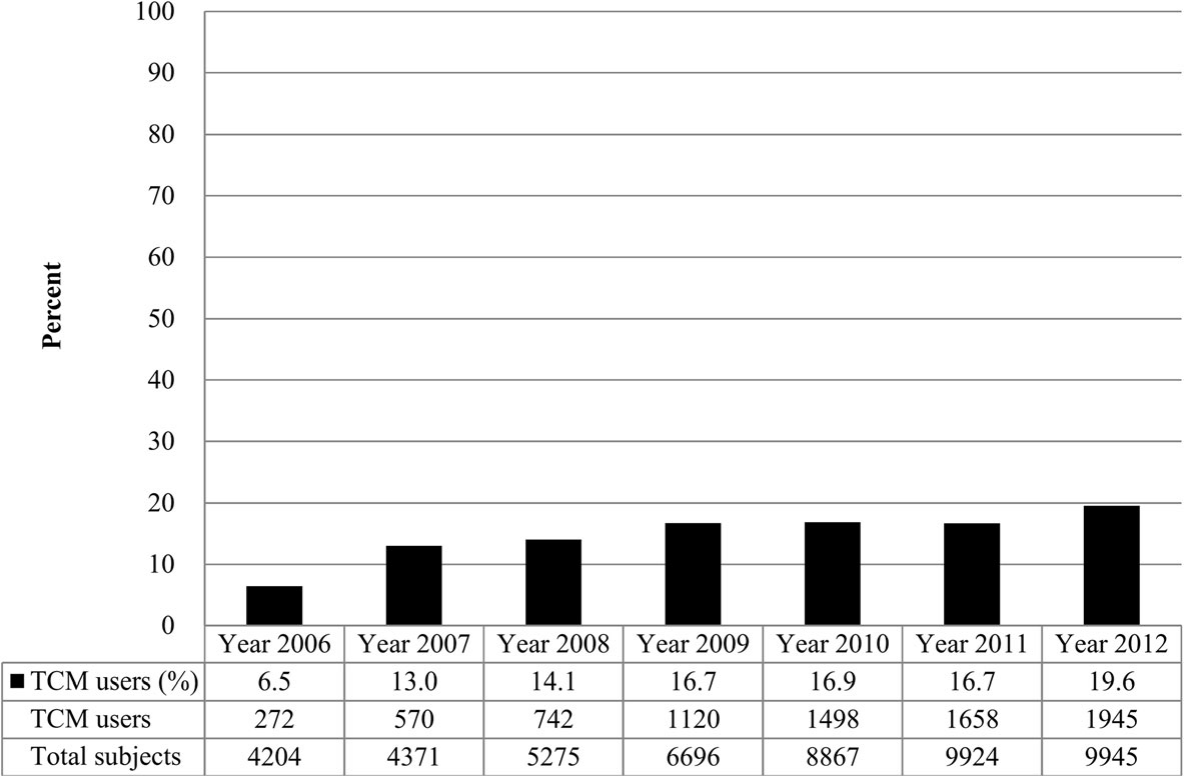
Rate of TCM use among inpatients with first-time stroke during hospitalization between 2006 and 2012

**Fig. 3:**
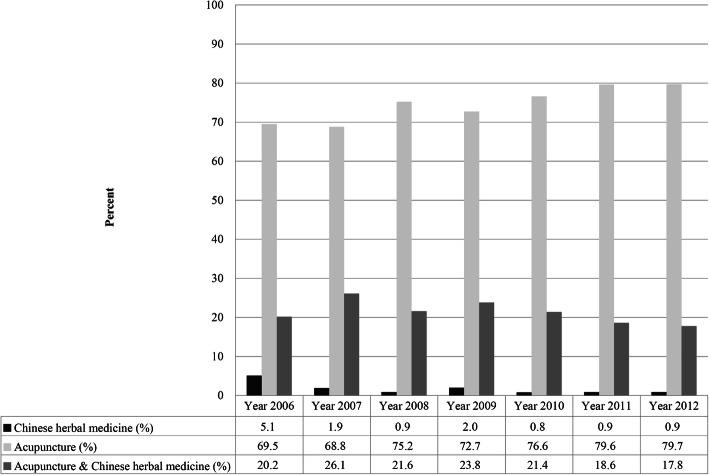
Rate of Chinese herbal medicine and acupuncture use among inpatients with first-stroke utilizing TCM during hospitalization in 2006–2012

The prevalence of TCM use among first-time stroke patients who were admitted to hospitals that joined the pilot scheme of TCM increased from 6.5% in 2006 to 19.6% in 2012 (Fig. 2). The annual rates of acupuncture use among first-time stroke inpatients ranged from 68.8 to 79.7%, while the rates of Chinese herbal medicine use ranged from 0.8 to 5.1%. The rates of combined acupuncture and Chinese herbal medicine use ranged from 17.8 to 26.1% (Fig. 3).

Table 1 lists the differences in characteristics between TCM users and TCM nonusers among first-time stroke inpatients between 2006 and 2012. TCM users tended to be younger (*P* < 0.001), have a longer hospital stay (*P* < 0.001), have higher stroke severity (*P* < 0.001), receive rehabilitation during hospitalization (*P* < 0.001), have experience with past TCM use (*P* < 0.001), and have higher insured amounts (*P* < 0.001) than nonusers.

**Table 1:** Characteristics of the patients with first-stroke admitted to hospitals participating in the TCM scheme

Characteristics	TCM users (*n* = 7455)	TCM non-users (*n* = 81,707)	*P*
Demographic factors			
Age (years)			< 0.001
≥ 65	3466 (46.5%)	44,111 (54.0%)	
45–64	3210 (43.1%)	31,065 (38.0%)	
< 45	779 (10.4%)	6531 (8.0%)	
Sex			0.142
Women	2912 (39.1%)	32,627 (39.9%)	
Men	4543 (60.9%)	49,080 (60.1%)	
Clinical factors			
Type of stroke			< 0.001
Hemorrhagic	2552 (34.2%)	21,235 (26.0%)	
Ischemic	4752 (63.8%)	59,433 (72.7%)	
Hemorrhagic & ischemic	151 (2.0%)	1039 (1.3%)	
Length of hospital stay for first-time stroke (days)			< 0.001
1–7	783 (10.5%)	43,133 (52.8%)	
8–14	1244 (16.7%)	18,442 (22.6%)	
15–28	1645 (22.1%)	11,211 (13.7%)	
≥ 29	3783 (50.7%)	8921 (10.9%)	
SSI score			< 0.001
≥ 20	1537 (20.6%)	9510 (11.6%)	
10–19	2697 (36.2%)	17,483 (21.4%)	
< 10	3221 (43.2%)	54,714 (67.0%)	
CCI score			< 0.001
≥ 3	127 (1.7%)	2321 (2.8%)	
1–2	2938 (39.4%)	31,313 (38.3%)	
0	4390 (58.9%)	48,073 (58.9%)	
Hospital accreditation level			< 0.001
Medical center	3866 (51.9%)	44,954 (55.0%)	
Regional hospital	3484 (46.7%)	36,076 (44.2%)	
Community hospital	105 (1.4%)	677 (0.8%)	
Rehabilitation during hospitalization for first-time stroke			< 0.001
No	474 (6.4%)	46,164 (56.5%)	
Yes	6981 (93.6%)	35,543 (43.5%)	
TCM treatment in the past 9 years before first-time stroke			< 0.001
No	2190 (29.4%)	26,352 (32.2%)	
Yes	5265 (70.6%)	55,355 (67.8%)	
Socioeconomic factors			
Urbanization residency level			< 0.001
1 (most urbanized)	1293 (17.4%)	15,215 (18.6%)	
2	2454 (32.9%)	25,119 (30.7%)	
3	1179 (15.8%)	13,929 (17.1%)	
4	1287 (17.3%)	13,737 (16.8%)	
5	286 (3.8%)	4007 (4.9%)	
6	471 (6.3%)	5448 (6.7%)	
7 (least urbanized)	485 (6.5%)	4252 (5.2%)	
Insured amount per month			< 0.001
< 576 USD	3272 (43.9%)	38,270 (46.8%)	
≥ 576 USD	4183 (56.1%)	43,437 (53.2%)	

Abbreviations: *TCM* Traditional Chinese medicine; *USD* United States dollar; *SSI* Stroke severity index; *CCI* Charlson-Deyo comorbidity index

Table 2 shows the results of the logistic regression analysis of factors that predict TCM use among first-time stroke inpatients. Patients aged < 45 years (OR, 1.58; 95% confidence interval [CI], 1.43–1.74) and 45–64 years (OR, 1.37; 95% CI, 1.29–1.45) were more likely to use TCM than patients aged ≥65 years (*P* < 0.001). Men were more likely to use TCM than women (OR, 1.08; 95% CI, 1.02–1.14; *P* = 0.007). Patients with ischemic stroke (OR, 1.20; 95% CI, 1.12–1.28; *P* < 0.001) were more likely to use TCM than those with hemorrhagic stroke. Patients hospitalized for first-time stroke for 8–14 days (OR, 2.08; 95% CI, 1.89–2.29), 15–28 days (OR, 4.10; 95% CI, 3.72–4.53), and ≥ 29 days (OR, 11.84; 95% CI, 10.72–13.08) were more likely to use TCM than those hospitalized for 1–7 days (*P* < 0.001). Patients with SSI scores of 0–9 (OR, 1.31; 95% CI, 1.20–1.44) and 10–19 (OR, 1.16; 95% CI, 1.08–1.26) were more likely to use TCM than those with SSI scores of ≥20 (*P* < 0.001). Patients with CCI scores of 0 (OR, 2.15; 95% CI, 1.77–2.61) and 1–2 (OR, 2.04; 95% CI, 1.68–2.48) were more likely to use TCM than those with CCI scores ≥3 (*P* < 0.001). Patients admitted to regional hospitals (OR, 1.41; 95% CI, 1.34–1.49) and community hospitals (OR, 6.22; 95% CI, 4.87–7.95) were more likely to use TCM than those admitted to medical centers (*P* < 0.001). Patients receiving rehabilitation during hospitalization for first-time stroke (OR, 9.16; 95% CI, 8.28–10.13; *P* < 0.001) were more likely to use TCM than those without rehabilitation. Patients who received outpatient TCM treatment in the past 9 years before first-time stroke (OR, 1.35; 95% CI, 1.28–1.43; *P* < 0.001) were more likely to use TCM than those without outpatient TCM treatment. Patients living in level 2 (OR, 1.18; 95% CI, 1.09–1.28), level 4 (OR, 1.21; 95% CI, 1.10–1.32), and level 7 urbanized areas (OR, 1.38; 95% CI, 1.22–1.56) were more likely to use TCM than those living in level 1 urbanized areas (*P* < 0.001). Patients with an insured amount of ≥576 USD per month (OR, 1.20; 95% CI, 1.14–1.27; *P* < 0.001) were more likely to use TCM than those with an insured amount of < 576 USD per month.

**Table 2:** Logistic regression analysis of potential factors affecting TCM use among inpatients with first-time stroke

Characteristics	Odds ratio	95% CI Lower	95% CI Upper	*P*
Age (years)				< 0.001
≥ 65 (reference group)	1.00			
45–64	1.37	1.29	1.45	
< 45	1.58	1.43	1.74	
Sex				0.007
Women (reference group)	1.00			
Men	1.08	1.02	1.14	
Type of stroke				< 0.001
Hemorrhagic (reference group)	1.00			
Ischemic	1.20	1.12	1.28	
Hemorrhagic & ischemic	1.18	0.97	1.44	
Length of hospital stay for first-time stroke (days)				< 0.001
1–7 (reference group)	1.00			
8–14	2.08	1.89	2.29	
15–28	4.10	3.72	4.53	
≥ 29	11.84	10.72	13.08	
SSI score				< 0.001
≥ 20 (reference group)	1.00			
10–19	1.16	1.08	1.26	
< 10	1.31	1.20	1.44	
CCI score				< 0.001
≥ 3 (reference group)	1.00			
1–2	2.04	1.68	2.48	
0	2.15	1.77	2.61	
Hospital accreditation level				< 0.001
Medical center (reference group)	1.00			
Regional hospital	1.41	1.34	1.49	
Community hospital	6.22	4.87	7.95	
Rehabilitation during hospitalization for first-time stroke				< 0.001
No (reference group)	1.00			
Yes	9.16	8.28	10.13	
TCM treatment in the past 9 years before first-time stroke				< 0.001
No (reference group)	1.00			
Yes	1.35	1.28	1.43	
Urbanization residency level				< 0.001
1 (reference group)	1.00			
2	1.18	1.09	1.28	
3	1.01	0.93	1.11	
4	1.21	1.10	1.32	
5	0.92	0.80	1.07	
6	1.06	0.94	1.20	
7 (least urbanized)	1.38	1.22	1.56	
Insured amount per month				< 0.001
< 576 USD (reference group)	1.00			
≥ 576 USD	1.20	1.14	1.27	

Abbreviations: *TCM* Traditional Chinese medicine; *CI* Confidence interval; *USD* United States dollar; *SSI* Stroke severity index; *CCI* Charlson-Deyo comorbidity index

## Discussion

This study demonstrated that various demographic, clinical, and socioeconomic factors may affect the use of TCM. TCM use among first-time stroke inpatients in Taiwan increased markedly from 2006 to 2012. Among TCM users, approximately 70% used acupuncture only, fewer than 6% used Chinese herbal medicine only, and approximately 20–25% used both acupuncture and Chinese herbal medicine. To the authors’ knowledge, this is the first nationwide study to report the prevalence of and factors associated with TCM use among first-time stroke inpatients in Taiwan.

Complementary and alternative medicine (CAM) is one of the most popular therapies among patients worldwide. In the US, approximately 50% of stroke survivors use CAM, with acupuncture being the most frequently used therapy [39]. TCM is usually considered a subdivision of CAM. In Korea, 54% of stroke patients used TCM, and 74% of them used TCM within 3 months of stroke onset [40]. In this study, the prevalence of TCM use among first-time stroke inpatients increased from 6.5% in 2006 to 19.6% in 2012; this three-fold increase over 7 years suggests the popularity of TCM compared with other traditional stroke treatments.

In Taiwan, TCM and medicine are two coexisting but independent healthcare systems under the NHI [20, 21]. However, medical doctors are responsible for treating and caring for stroke inpatients. As mentioned in the introduction, before 2006, there was no TCM scheme [12, 22]. Stroke inpatients who wished to receive TCM therapy during hospitalization had to pay for it themselves or choose outpatient TCM therapy after discharge. However, outpatient TCM therapy is inconvenient for stroke patients, and they may discontinue treatment due to mobility, transportation, and economic issues. The implementation of the TCM scheme provided an additional and alternative therapy for acute and subacute stroke patients during hospitalization.

The recommendation of first-line physicians or surgeons who provide care for stroke patients may influence the patients’ use of TCM [40] because these professionals have the power to decide whether to recommend TCM treatment or not. However, first-line physicians’ or surgeons’ perceptions of TCM treatment depends on various factors [41], such as their knowledge about the effects of TCM on stroke and hospital policies. The widespread use of TCM is possible, although an increase in TCM use among first-time stroke inpatients was observed in the present study. The next step is to validate the effects of TCM on the recovery of first-time stroke patients. This will enhance physicians’ or surgeons’ knowledge about the outcomes of therapy, and thus, they may be more receptive to the use of TCM in stroke patients.

In this study, TCM users were more likely to use rehabilitation than TCM nonusers, and the prevalence of TCM use in patients who received rehabilitation was 9.2 times higher than in those who did not. Using rehabilitation during hospitalization for first-time stroke is a strong predictor of TCM use, as shown in a previous study [22]. In Taiwan, stroke rehabilitation therapy is covered by the NHI [42]; therefore, first-time stroke inpatients commonly receive rehabilitation therapy. In Taiwan, approximately 43.6% of first-time stroke survivors received rehabilitation during their hospitalization for first-time stroke [35]. Clinically, most patients started receiving rehabilitation when their neurological status and vital signs stabilized [43]. The researchers inferred that the suitable timing of TCM use was similar to that of rehabilitation use in first-time stroke patients. The overlapping period of using TCM and rehabilitation may contribute to positive treatment results.

In this study, TCM use was higher among patients hospitalized for longer periods, especially over 28 days. This result was compatible with that of a previous study [22]. If patients stay in the hospital longer for inpatient rehabilitation, they may obtain more information about TCM and understand it better, thereby increasing their willingness to use TCM. However, a longer hospital stay may depend on different factors, such as the patient’s medical condition, the waiting time to transfer to the next medical service to proceed with rehabilitation, and the NHI policy. More studies are needed to analyze these factors in the future.

Patients admitted to regional and community hospitals used TCM more frequently than patients admitted to medical centers. According to this study, among the hospitals joining the TCM scheme from 2006 to 2012, 27.5% were medical centers, 65% were regional hospitals, and the remaining 7.5% were community hospitals. The odds of using TCM were 6.2 times higher in patients admitted to community hospitals than in those admitted to medical centers. However, one article by Chang et al. illustrated that patients admitted to medical centers with a TCM department were more likely to use TCM in Korea [26]. These differences in TCM use between the two studies may be attributed to the different healthcare systems and hospital policies in Korea and Taiwan. In Korea, patients can easily consult TCM doctors at academic medical centers [26]. In Taiwan, although most hospitals have TCM departments, they offer only outpatient services. The researchers considered that most medical centers in Taiwan may have less motivation to encourage their TCM departments to join the TCM scheme because their hospital revenues are always higher than regional and community hospitals. Instead, regional and community hospitals may be more willing to join the TCM scheme because they pursue more profit (i.e., more claims to the NHI).

Liao et al.’s [24] and Weng et al.’s [25] studies showed that TCM use was higher among stroke patients living in urban areas than among those living in rural areas. The present study showed that TCM use was higher among patients living in level 2, 4, and 7 urbanized areas than among those living in level 1 urbanized areas. Although the results did not show the same differences in all levels (compared with level 1), the findings herein still demonstrated that first-time stroke patients living in rural areas are more willing to use TCM. The differences in the findings among our study, Liao et al.’s study [24], and Weng et al.’s study [25] may be because they enrolled patients using outpatient TCM services for 1 year after stroke, whereas we only enrolled patients using TCM during their hospitalization for first-time stroke. In Taiwan, highly urbanized areas have a high density of TCM doctors offering outpatient services [44]. This easy access to outpatient TCM services may influence the healthcare-seeking behaviors of first-time stroke patients after hospital discharge.

Stroke patients have more comorbidities that require professional attention [45]. Physical disability may be due to comorbid diseases [45, 46], which may prevent them from using TCM. Wei et al. found that higher stroke severity and more comorbidities [22] were positively associated with increased TCM use among stroke inpatients. However, our findings showed that lower stroke severity (lower SSI scores) and fewer comorbidities (lower CCI scores) may increase TCM use among first-time stroke inpatients. The difference between our findings and those of Wei et al. may be due to differences in stroke types and hospital types. Wei et al. [22] enrolled 4064 inpatients with ischemic stroke from a medical center in Taiwan, but we enrolled 89,162 inpatients with first-time ischemic and hemorrhagic stroke admitted to hospitals with different accreditation levels in Taiwan. We considered that first-time stroke patients with higher stroke severity and more comorbidities may need intensive care for more days to stabilize their medical condition during hospitalization. Due to the limited days of hospitalization, it is less possible to receive TCM while under intensive care for medical problems related to greater severity and more comorbidities.

The researchers found that TCM use was higher among ischemic stroke inpatients than among those with hemorrhagic stroke. Weng et al. [25] also showed similar trends in outpatient TCM use among patients with different types of stroke. In the literature, acupuncture enabled functional recovery in stroke patients through several mechanisms, such as brain reorganization [47], the regulation of neurochemical release in ischemic stroke patients [48], and the inhibition of TNF-α/NFκB expression in a rat model of hemorrhagic stroke [49]. Chinese herbal medicine is a popular choice for treating stroke [14]. It is considered to have both in vitro and in vivo angiogenic effects in the context of ischemic stroke [50], neuroprotective effects in mice with ischemic stroke via the modulation of molecular targets or genes [51], and improved blood perfusion in the brains of hemorrhagic stroke patients [52]. Although there is more evidence of the benefits of acupuncture or Chinese herbal medicine on the ischemic brain than on the hemorrhagic brain, further studies are needed to prove that TCM is more beneficial in ischemic stroke than in hemorrhagic stroke.

A number of physicians, patients, and their families are concerned about the bleeding tendency and side effects of TCM. However, evidence shows that acupuncture is safe and may only cause minor adverse events, such as pain, dizziness, and fainting [10]. A study applying the Cochrane systematic review methods to 59 kinds of Chinese herbal medicines [9] found that these medicines are relatively free of major adverse effects and are nontoxic to patients with ischemic stroke. However, certain Chinese herbal medicines may increase the risk of bleeding when used in combination with anticoagulants [53]. It must be noted that Chinese herbal medicine may have side effects when used in combination with other medicines [54]. Hence, Chinese herbal medicine, similar to medicine, is relatively safe if used on the basis of therapeutic principles and correct diagnosis. Moreover, adverse pharmacological effects can be avoided if drug compatibility is assessed and suitable drug administration methods are followed [54].

This study had limitations common to retrospective database analyses. First, the diagnoses were sourced from the NHIRD based on ICD-9-CM codes, which may be less accurate than prospective diagnoses. Second, we collected medical claims data that lacked additional information regarding the factors that may influence TCM use. Third, because not all hospitals had TCM services and not all hospitals with TCM services joined the TCM scheme, the prevalence of TCM use may not represent the entire population of first-time stroke patients in Taiwan. Despite these limitations, this study provides a clear profile of the use of pilot scheme-supported TCM among first-time stroke inpatients in Taiwan. Future prospective studies focusing on collecting information on the diagnoses and potential factors that influence TCM use are warranted to address the existing knowledge gap. More studies are needed to provide evidence of better outcomes after TCM use in first-time stroke patients.

## Conclusions

Previous studies have shown that TCM is beneficial for reducing the complication rate, reducing the mortality rate and enhancing the quality of life of stroke patients. The TCM pilot scheme is a quick and convenient way to provide TCM to willing first-time stroke patients. The overall trend showed positive growth and greater acceptance of TCM use among first-time stroke patients. This study clearly demonstrated that the important predictors for TCM use among first-time stroke inpatients in Taiwan were age, sex, area of residence, income status, length of hospital stay, hospital accreditation level, type and severity of stroke, presence of comorbidities, receiving rehabilitation, and previous experience with outpatient TCM services. These findings could provide information on TCM use among first-time stroke inpatients to first-line medical staff and hospital policy makers.

## Data Availability

The datasets used and/or analyzed during the current study are available from the corresponding author on reasonable request.

## Abbreviations

TCM: Traditional Chinese medicine
NHI: National health insurance
NHIRD: National health insurance research database
ICD-9-CM: International classification of diseases, 9th edition, clinical modification
SSI: Stroke severity index
CCI: Charlson-Deyo comorbidity index
CAM: Complementary and alternative medicine
